# *P. granatum* Peel Polysaccharides Ameliorate Imiquimod-Induced Psoriasis-Like Dermatitis in Mice *via* Suppression of NF-κB and STAT3 Pathways

**DOI:** 10.3389/fphar.2021.806844

**Published:** 2022-01-28

**Authors:** Haiming Chen, Cheng Wang, Bin Tang, Jingjie Yu, Yue Lu, Junhong Zhang, Yuhong Yan, Hao Deng, Ling Han, Shaoping Li, Chuanjian Lu

**Affiliations:** ^1^ State Key Laboratory of Dampness Syndrome of Chinese Medicine, The Second Affiliated Hospital of Guangzhou University of Chinese Medicine, Guangzhou, China; ^2^ Guangdong Provincial Key Laboratory of Clinical Research on Traditional Chinese Medicine Syndrome, Guangzhou, China; ^3^ Guangdong Clinical Research Center for Dermatosis in Chinese Medicine, Guangzhou, China; ^4^ Guangdong-Hong Kong-Macau Joint Lab on Chinese Medicine and Immune Disease Research, Guangzhou University of Chinese Medicine, Guangzhou, China; ^5^ State Key Laboratory of Quality Research in Chinese Medicine, Institute of Chinese Medical Sciences, University of Macau, Macao SAR, China; ^6^ Joint Laboratory of Chinese Herbal Glycoengineering and Testing Technology, University of Macau, Macao SAR, China

**Keywords:** *P. granatum* peel polysaccharides, anti-inflammation, skin barrier, imiquimod-induced psoriasis, NF-κB, STAT3

## Abstract

Psoriasis is a chronic and refractory inflammatory and autoimmune-mediated cutaneous disease affecting approximately 2%–3% of the global population. Most of the current therapies could relieve symptoms rapidly, while the side effects cannot be negligible. Hence, it is urgent to explore much safer and more effective treatments. In the current work, we evaluated the potential beneficial effect of *Punica granatum* peel polysaccharides (PPPs) in an imiquimod-elicited psoriasis-like mouse model and unraveled their mechanism of action. Firstly, PPPs were isolated from *P. granatum* peels, and then the molecular weight was determined and monosaccharide analysis was performed. The results revealed that PPPs significantly ameliorated psoriasis-like skin lesions and reduced the Psoriasis Area and Severity Index (PASI) scores and transepidermal water loss (TEWL). PPPs also attenuated the expressions of CD3 and Ki67 in psoriasis-like mouse skin and suppressed the serum or skin levels of pro-inflammatory cytokines, such as tumor necrosis factor alpha (TNF-α), interleukin 6 (IL-6), IL-1β, IL-8, IL-17, and IL-23. Moreover, PPPs were able to upregulate the mRNA and protein expressions of aquaporin-3 (AQP3) and filaggrin (FLG) in the skin of mice. In addition, PPPs inhibited the NF-κB and STAT3 signaling pathways. Overall, these results indicated that PPPs ameliorated the symptoms of psoriasis through inhibition of the inflammatory cytokines by suppressing the NF-κB and STAT3 signaling pathways and improved skin barrier protection *via* enhancing AQP3 and FLG. These observations potentially contribute to providing theoretical and experimental evidence for the clinical application of PPPs for psoriasis.

## Introduction

Psoriasis is a refractory cutaneous ailment that is closely related to immune-mediated inflammation. The pathological manifestations of psoriasis include immune cell infiltration and abnormal keratinocyte proliferation in the epidermis (Greb et al., 2016). Approximately 2%–3% of the global population suffers from psoriasis (Zhou et al., 2020). The prevalence of psoriasis in adults is higher than that in children. Poor quality of life has been observed in most patients with psoriasis (Michalek et al., 2017). The primary treatment options for psoriasis are immunosuppression and biological agents, although these therapies can only relive symptoms for a short time. Relapse may occur when the treatment is ceased. Long-term use of these therapies is likely to cause side effects, including infection and liver toxicity (Chen et al., 2018; Mahil and Smith, 2019). Therefore, research and development of innovative effective and safe anti-psoriatic drugs is still an urgent need. Recently, the application of Chinese herbal medicines in psoriasis treatment has been widely reported (Di et al., 2021; Lu et al., 2021b; Lv et al., 2021; Yao et al., 2021).

Pomegranate peel is the dried peel of *Punica granatum* L., a plant in the pomegranate family. In traditional Chinese medicine, the functions of pomegranate peel include hemostasis, sedation, and antibacterial. Modern pharmacological studies have found that pomegranate peel polysaccharide has a significant immunomodulatory effect and good antioxidant activity in immunosuppressed mice induced by cyclophosphamide (Wu et al., 2019). In addition, it showed a significant preventive effect in a liver injury model induced by CCl_4_ (Zhai et al., 2018b). An *in vitro* experiment has also shown that many parts of *P. granatum* peel polysaccharides (PPPs) have strong reducing activities and good scavenging activities to 2,2-diphenyl-1-picrylhydrazy (DPPH) free radicals, hydroxyl groups, and superoxide anions (Zhai et al., 2018a).

However, the protective effect of pomegranate peel polysaccharides on the skin lesions of an imiquimod (IMQ)-elicited psoriasis-like animal model has not been reported previously. In the present work, we explored the protective effects of PPPs in a psoriatic mice model and probed their potential biological mechanism.

## Materials and Methods

### Chemical and Reagents

Compound dexamethasone acetate cream (DXA) was obtained from China Resources Sanjiu Medical and Pharmaceutical Co., Ltd. (Shenzhen, China). Imiquimod cream was purchased from Sichuan Mingxin Pharmaceutical Co., Ltd. (Sichuan, China). *P. granatum* peels were obtained from fruit purchased from Kangmei Pharmaceutical Co., Ltd. (Guangdong, China). The enhanced chemiluminescence (ECL) reagent was obtained from Millipore (Billerica, MA, USA). d-Fucose (Fuc), d-xylose (Xyl), d-galacturonic acid (GalA), d-galactose (Gal), d-glucose (Glc), d-mannose (Man), l-rhamnose (Rha), d-arabinose (Ara), d-glucuronic acid (GlcA), and d-fructose (Fru) were obtained from Sigma (St. Louis, MO, USA). Deionized water was prepared using a Millipore MilliQ Plus system (Millipore, Bedford, MA, USA). All the other reagents were of analytical grade.

### Animals

BALB/c mice (20 ± 2 g) were from the Center of Laboratory Animals of Southern Medical University (Guangzhou, China) and were offered free access to food and water within a specific pathogen-free (SPF) environment. Ethics approval of all animal experiments was obtained from the Animal Experimental Ethics Committee of Guangdong Provincial Hospital of Chinese Medicine (no. 2019012).

### Isolation of Polysaccharides

*P. granatum* peels were crushed into powder and the liposoluble ingredients removed with petroleum ether (1:5, *w*/*v*). Then, the skimmed materials were extracted with water (1:12, *w*/*v*) twice and the aqueous extracts collected, filtered, and evaporated. Subsequently, the obtained mixtures were precipitated by the addition of ethanol to 50% concentration for 24 h at 4°C. Precipitation was gathered by centrifuging at 5,000 rpm for 30 min. Lastly, the sediment was dialyzed (3,500 Da molecular weight cutoff, MWCO) and finally lyophilized to obtain crude PPPs.

### HPSEC–MALLS/RID Analysis

Based on a previously reported method (Cheong et al., 2015; Deng et al., 2018), the content and the molecular weight of PPPs were measured using high-performance size exclusion chromatography with multi-angle laser light scattering/refractive index detector (HPSEC–MALLS/RID), which was composed of an Agilent 1200 series LC-DAD system (Agilent Technologies, Palo Alto, CA, USA), a RID (Optilab T-rEX; Wyatt Technology Co., Santa Barbara, CA, USA), and a multi-angle laser light scattering detector (DAWN HELEOS; Wyatt Technology Co.) equipped with a He–Ne laser (*λ* = 658 nm), based on d*n/*d*c* (0.15 ml/g). An appropriate amount of PPPs was dissolved in the mobile phase (0.9% NaCl water solution) to prepare the concentration of 2.0 mg/ml. The solution was filtered through a 0.45-μm membrane prior to analysis and 100 μl sample solution was injected. The separation was carried out on two columns in series, TSK-GEL G5000PWXL (300 mm × 7.8 mm, i.d.) and TSK-GEL G3000PWXL (300 mm × 7.8 mm, i.d.), with a flow rate of 0.5 ml min^−1^ at 35°C. Data collection and analysis were carried out using Astra software (version 6.0.2, Wyatt Technology Corp.).

### Monosaccharide Analysis

#### Sample Preparation

The sample of polysaccharides in *P. granatum* peel (2.0 mg) was subjected to hydrolysis with 1.0 ml trifluoroacetic acid (TFA, 2.0 M) at 105°C for 6 h. Then, the reaction mixtures were washed with methanol and dried with a nitrogen evaporator 3 times to remove the TFA residue. Subsequently, 1.0 ml pure water was added for reconstitution and diluted 10 times, and then filtered using a 0.45-μm filter before analysis.

### HPAEC–PAD Analysis

According to the method reported in a previous study (Alyassin et al., 2020), compositional monosaccharide analysis of PPPs was carried out with a Dionex ICS-3000 Ion Chromatography System (Dionex Corporation, Sunnyvale, CA, USA) containing an AS50 autosampler, an ICS-3000 dual pump, and an ICS-3000 DC. Chromeleon® (6.8) software was utilized to analyze the data. The samples and mix standard solutions were separated by a CarboPac PA200 (3 mm × 250 mm) with a CarboPac PA20 guard (3 mm × 30 mm) column at 25°C, and the flow rate was set at 0.5 ml/min. The injection volume was set at 5 μl and subjected to elution by a gradient prepared with deionized water (eluent A), 10 mM sodium hydroxide (eluent B), and 0.5 M sodium acetate (eluent C). Eluent B was constant (12%) and eluent A varied from 88% at 30 min to 43% at 22 min. This proportion was maintained constant until 25 min, when the initial conditions were recovered. An external standard method with a mixture consisting of 10 monosaccharide reference substances (Glc, Rha, Xyl, Ara, Fuc, GalA, GlcA, Gal, Fru, and Man) in 20 μg/ml was utilized to investigate the compositional monosaccharides of PPPs. All analyses were performed in duplicate.

### Administration of Test Articles

BALB/c mice were randomly allocated into 5 groups (*n* = 6): control, vehicle, DXA (1 mg kg^−1^ day^−1^), low-dose PPP group (0.25 g/ml, PPPs-L), and high-dose PPP group (0.5 g/ml, PPPs-H). The control group consisted of normal mice devoid of treatment. Mice in the PPP and DXA groups were topically treated with PPPs and DXA, respectively, and distilled water was topically administered to the control and vehicle groups. Topical use of IMQ cream was applied to mice in the vehicle and treatment groups in order to induce psoriasis for 7 consecutive days.

### Imiquimod-Induced Psoriasis-Like Mouse Model

On the basis of our previous study (Chen et al., 2020), BALB/c mice were subjected to topical treatment with 5% IMQ cream (62.5 mg), which was applied on a shaved area (3 cm × 2.5 cm) on the back for 7 consecutive days. The PASI, measured on the 7th day, is an assessment tool combining (Lu et al., 2021a) skin erythema, scaling, and thickness, which was employed to evaluate the severity grade of psoriasis-like lesions. Transepidermal water loss (TEWL) was measured on the back skin of the psoriasis-like mouse model with VAPOSCANAS-VT100 (Tokyo, Japan).

### Histological Evaluation and Immunohistochemistry

The mouse skin samples were removed and subjected to 24-h fixation in 4% paraformaldehyde and embedded in paraffin. Then, the skin samples in paraffin were cut into sections (5 μm) and hematoxylin and eosin (H&E) staining was applied for histological evaluation. For immunohistochemical staining, the slides were subjected to incubation overnight at 4°C with specific primary antibodies against Ki67 (1:1,000; Servicebio, Wuhan, China) and CD3 (1:1,000; Abcam, Waltham, MA, USA). Subsequently, the sections were incubated with biotinylated secondary antibodies (1:1,000; Abcam) for 1 h at 25°C, followed by diaminobenzidine staining and hematoxylin counter staining.

### Measurements of TNF-*α*, IL-6, IL-1*β*, AQP3, and FLG mRNA Expression *via* RT-PCR

Furthermore, the mRNA levels of tumor necrosis factor alpha (TNF-*α*), interleukin 6 (IL-6), IL-1*β*, aquaporin-3 (AQP3), and filaggrin (FLG) in the skin samples were assessed *via* RT-PCR. Total mRNA was extracted from the skin samples *via* the TRIzol reagent, and mRNA was reversely transcribed to cDNA. The primer sequences are tabulated in Table 1. The relative mRNA expression of inflammatory cytokines *versus* β-actin was evaluated using an ABI 7500 Fast Real-Time PCR System (Thermo Fisher Scientific, Waltham, MA, USA).

**TABLE 1:** Primer sequences of target genes

Target gene		Primer sequence (5′→3′)
*TNF-α*	Forward	ACT​GAT​GAG​AGG​GAG​GCC​AT
	Reverse	CCG​TGG​GTT​GGA​CAG​ATG​AA
*IL-6*	Forward	TTC​TTG​GGA​CTG​ATG​CTG​GT
	Reverse	CCT​CCG​ACT​TGT​GAA​GTG​GT
*IL-1β*	Forward	TGC​CAC​CTT​TTG​ACA​GTG​ATG
	Reverse	AAG​GTC​CAC​GGG​AAA​GAC​AC
*AQP3*	Forward	GCT​TTT​GGC​TTC​GCT​GTC​AC
	Reverse	TAG​ATG​GGC​AGC​TTG​ATC​CAG
*FLG*	Forward	ATG​TCC​GCT​CTC​CTG​GAA​AG
	Reverse	TGG​ATT​CTT​CAA​GAC​TGC​CTG​TA
*β-actin*	Forward	GTG​ACG​TTG​ACA​TCC​GTA​AAG​A
	Reverse	GCC​GGA​CTC​ATC​GTA​CTC​C

### Measurements of TNF-α, IL-6, IL-17, IL-23, and IL-8 Levels with ELISA

Meanwhile, the levels of TNF-α, IL-6, IL-17, IL-23, and IL-8 were assessed in the mouse serum with commercially available ELISA kits (Meimian, Jiangsu, China). The absorbance was read at 450 nm using a microplate spectrophotometer (Multiskan GO; Thermo Fisher Scientific).

### Western Blotting Analysis

In addition, the protein expressions of p-NF-κB, NF-κB, p-STAT3, STAT3, AQP3, and FLG were evaluated *via* Western blotting. Firstly, RIPA lysis buffer was used to extract total protein from mouse skin samples, and a BCA assay kit (Thermo Fisher Scientific) was employed to measure the protein concentrations. An equal amount of protein from each sample was loaded to SDS-PAGE and transferred to PVDF membranes. The membranes were then blocked with 5% (*w*/*v*) skimmed milk in TBS-T containing 0.1% Tween-20 at room temperature for 2 h and subsequently incubated with specific primary antibody against p-NF-κB (p65) [1:1,000; Cell Signaling Technology (CST), Danvers, MA, USA], NF-κB (p65) (1:1,000; CST), p-STAT3 (1:1,000; CST), STAT3 (1:1,000; CST), AQP3 (1:1,000; Cohesion Biosciences, London, UK), FLG (1:1,000; Cohesion Biosciences), and *β*-actin (1:1,000; CST) at 4°C overnight. Subsequently, the membranes were washed with TBS-T and blotted with the corresponding secondary antibody (1:1,000; CST) for 1 h. Finally, the immunoreactive band was monitored using an enhanced chemiluminescence (ECL) method. ImageJ software (NIH, Bethesda, MD, USA) was adopted to quantitate the band intensity, and β-actin was used as the loading control.

### Statistical Analysis

Data were analyzed with one-way analysis of variance (ANOVA) followed by Dunnett’s test and expressed as the mean ± standard deviation (SD). Statistically significant differences were identified when *p* < 0.05. Statistical analysis was performed using GraphPad Prism 5.0 (GraphPad Software, La Jolla, CA, USA).

## Results

### Molecular Parameter Analysis of Polysaccharides in *P. granatum* Peel

**FIGURE 1:**
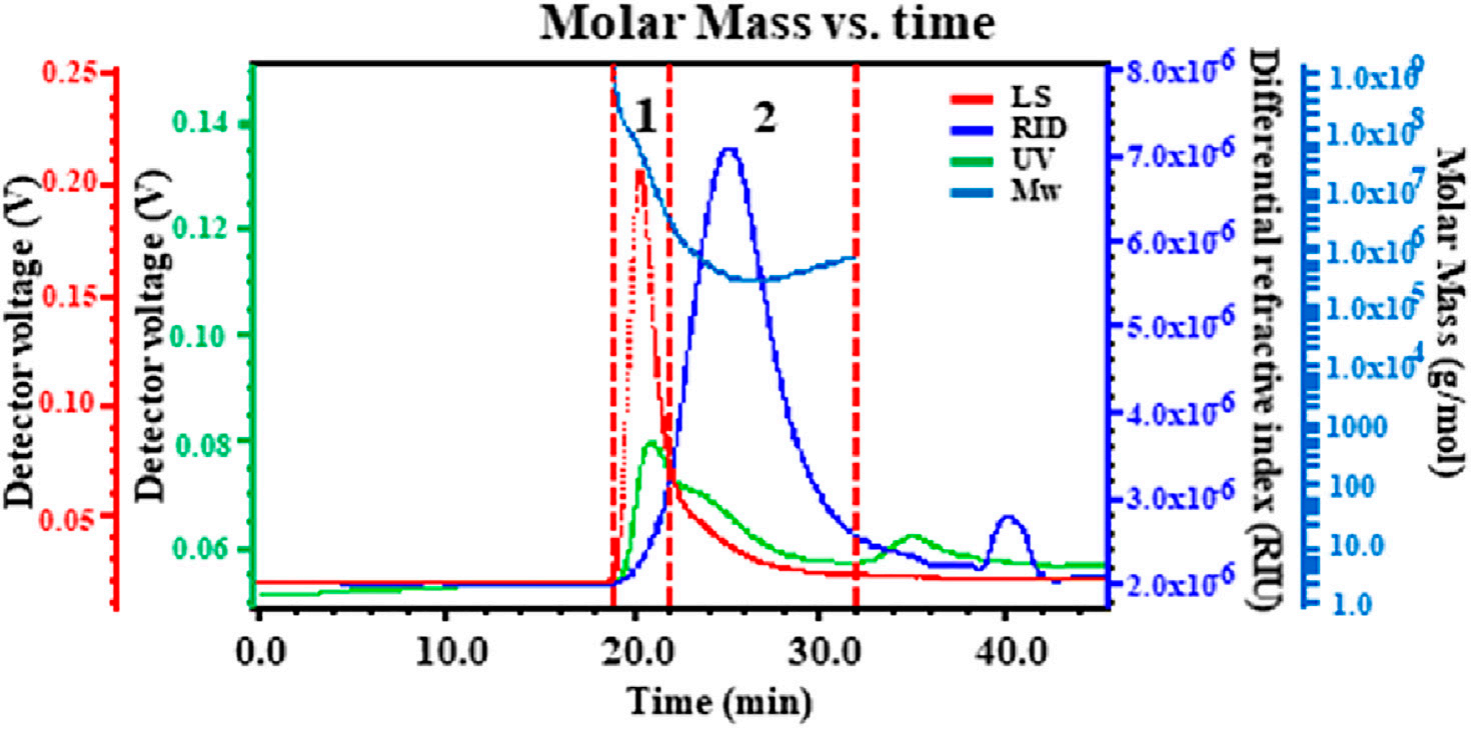
Typical high-performance size exclusion chromatography (HPSEC) of *Punica granatum* peel polysaccharides (PPPs).

The biological activities of polysaccharides from a natural resource are likely influenced by their molecular weight. The performance of HPSEC–MALLS/RID on the determination of the molecular weights and the molecular weight distribution of polysaccharides was excellent. Therefore, the molecular weights (*M*_w_), radius of gyrations (*R*_g_), polydispersity (*M*_w_/*M*_n_), and molecular weight distributions of PPPs were investigated using HPSEC–MALLS/RID. The chromatograms were found to be divided into two peaks (Figure 1). UV detection (green lines) indicated that PPPs contained proteins. The *M*_w_ of peaks 1 and 2 of PPPs were 2.272 × 10^7^ and 4.110 × 10^5^, respectively. The *M*_w_/*M*_n_ values were 2.391 and 1.479 and the *R*_g_ values were 33.9 and 35.9, respectively.

### Monosaccharide Composition of Polysaccharides in *P. granatum* Peel

**FIGURE 2:**
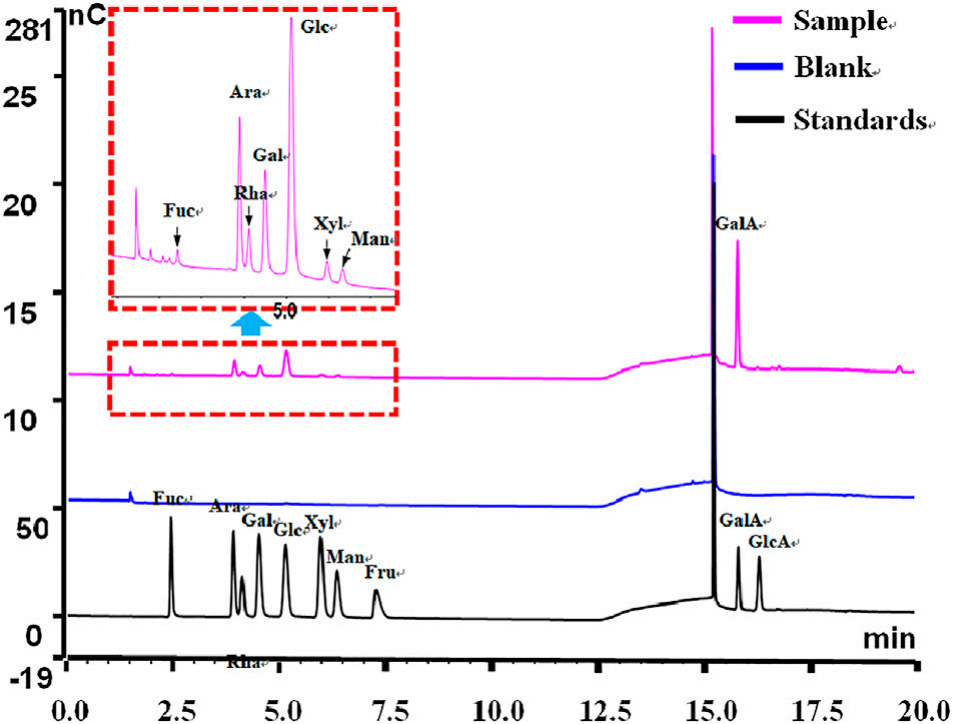
Typical high-performance anion exchange chromatography (HPAEC) of *Punica granatum* peel polysaccharides (PPPs).

High-performance anion exchange chromatography/pulse amperometric detection (HPAEC–PAD) analysis has been commonly used for the identification and quantitative determination of monosaccharides due to the advantages of speed, high specificity, and high sensitivity, as well as sample derivatization generally not being required (Templeton et al., 2012; Wang et al., 2014). The monosaccharide composition of PPPs was determined with HPAEC-PAD. By comparing to standard chromatograms, the monosaccharide peaks were confirmed. The results showed that the monosaccharide composition of PPPs consisted of Fuc, Ara, Rha, Gal, Glc, Xyl, Man, and GalA (Figure 2), and their molar ratio was 1.00:13.38:7.21:7.58:22.39:1.62:1.86:131.63, respectively. The main neutral monosaccharides were Ara, Rha, and Glc, as well as minor Fuc. The content of GalA was high (17.69%) in the monosaccharide composition of PPPs. The results of the monosaccharide composition analysis were congruent with a preceding report (Shakhmatov et al., 2019).

### *P. granatum* Peel Polysaccharides Attenuate Imiquimod-Elicited Psoriasis-Like Skin Lesion in Mice

**FIGURE 3:**
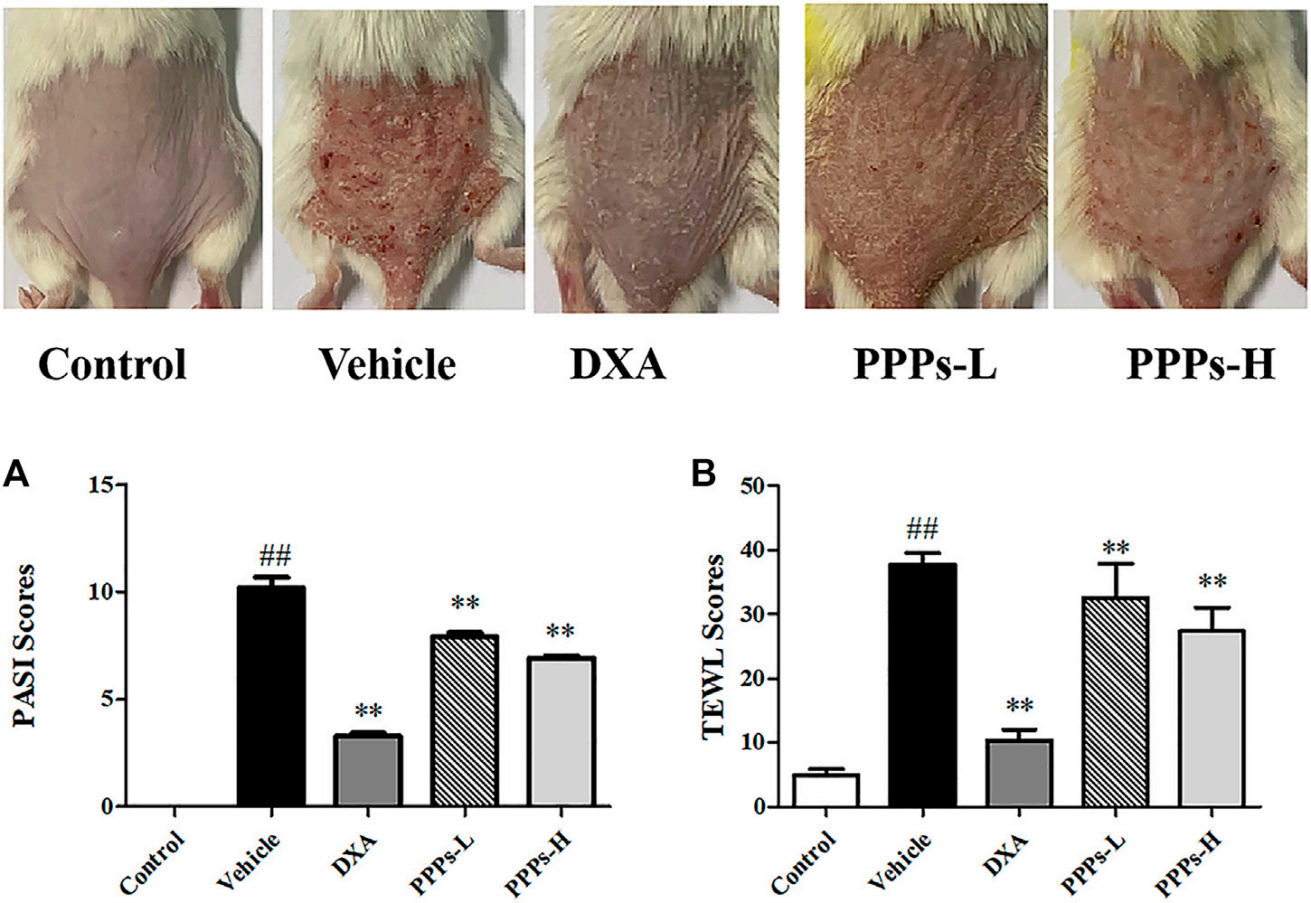
*Punica granatum* peel polysaccharides (PPPs) ameliorated imiquimod (IMQ)-induced psoriasis in BALB/c mice. Representative photographs of the dorsal skin in the IMQ-elicited psoriasis mouse model post-IMQ treatment without or with PPPs. **(A)** Psoriasis Area and Severity Index (PASI) scores of the skin lesions in mice with IMQ-elicited psoriasis post-treatment. **(B)** Transepidermal water loss (TEWL) of the skin lesions in IMQ-induced psoriasis-like mice. Data are presented as the mean ± SD (*n* = 6). ^#^ *p* < 0.05, ^##^ *p* < 0.01 *vs*. the control group; *p < 0.05, **p < 0.01 *vs*. the vehicle group. *PPPs-L*, low-dose PPPs; *PPPs-H*, high-dose PPPs.

In this study, the beneficial effects of PPPs were evaluated using an IMQ-elicited psoriasis mouse model. Marked epidermal scaling, erythema, and inflammatory infiltrates were observed on the dorsal skin after topical use of IMQ in comparison to the control group (Figure 3). The overall skin lesions of mice and the average PASI scores were notably decreased post-treatment with PPPs or DXA in comparison to the vehicle group (Figure 3A). TEWL is a known indicator that can serve to assess the function of the skin barrier (Agren et al., 2010). In our study, the TEWL was dramatically increased after exposure to IMQ (Figure 3B). However, PPPs could reverse the TEWL compared with the vehicle group, which indicated that PPPs were able to repair the skin barrier in the IMQ-elicited psoriasis mouse model.

### Histological Analysis

**FIGURE 4:**
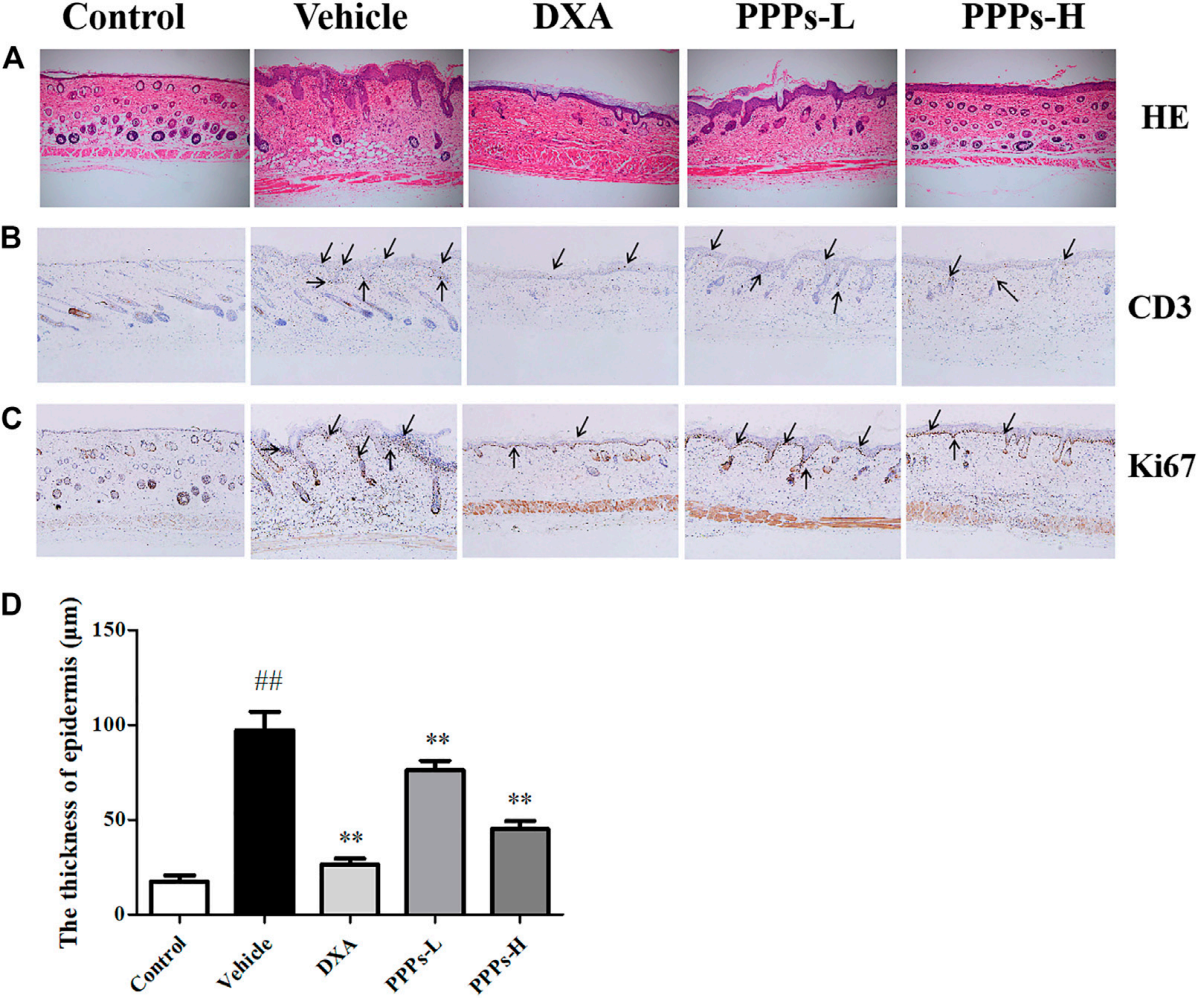
Histological analysis and immunohistochemistry assay. Different treatments were administrated to mice. H&E staining **(A)** of the dorsal skin lesion in different groups. Immunohistochemical photographs of CD3 **(B)** and Ki67 **(C)** staining (magnification, ×100) of the dorsal skin in control or psoriatic mice post-treatment. The black arrows indicate the CD3 and Ki67 positive location. ##*p* < 0.01 compared with the control group; **p < 0.01 compared with the vehicle group. *PPPs-L*, low-dose PPPs; *PPPs-H*, high-dose PPPs.

H&E staining was carried out for histological examination of the lesion skins post-treatment with PPPs. As depicted in Figures 4A,D, the tissue slide of mouse skin in the control group showed a normal smooth epidermis devoid of any inflammation or lesion. However, noteworthy pathological variations characteristic of accentuated acanthosis, hyperkeratosis of the epidermis, and abnormal inflammatory infiltrates were shown in the mouse skin of the vehicle group. In contrast, the administration of PPPs led to a much smoother epidermis with attenuated parakeratosis and ameliorated epidermal thickening in comparison to the vehicle counterpart.

### Effect of *P. granatum* Peel Polysaccharides on the Expressions of Ki67 and CD3 by Immunohistochemistry

The translational expressions of Ki67 and CD3 were detected in mouse skin since hyperproliferation and inflammation infiltration are crucial to psoriasis (Li et al., 2020). It was observed from the results (Figures 4B, C) that the expressions of Ki67 and CD3 in vehicle mice were markedly higher than those in the control counterpart. However, the expression levels of Ki67 and CD3 were noticeably suppressed by treatment with PPPs.

### *P. granatum* Peel Polysaccharides Reduce Pro-Inflammatory Cytokines in the Skin and Serum of Imiquimod-Treated Psoriatic Mice

**FIGURE 5:**
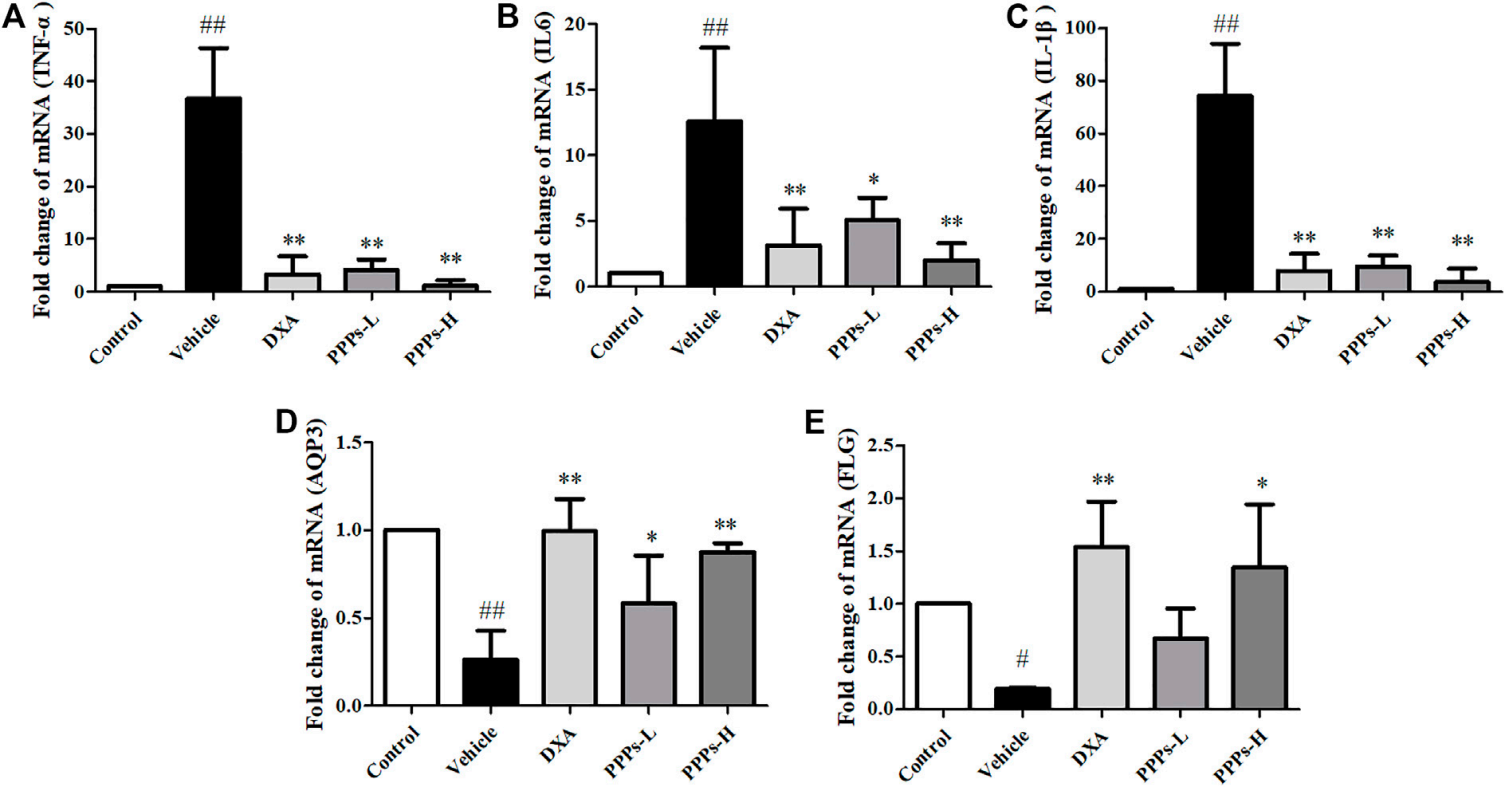
Effect of *Punica granatum* peel polysaccharides (PPPs) on the mRNA expressions of inflammatory cytokines, aquaporin-3 (AQP3), and filaggrin (FLG) in imiquimod (IMQ)-elicited psoriasis. Mice were treated with dexamethasone (DXA) and PPPs and topically administered with IMQ. The mRNA expressions of TNF-α **(A)**, IL-6 **(B)**, IL-1β **(C)**, AQP3 **(D)**, and FLG **(E)** in the skin were measured by RT-PCR. Data are shown as the mean ± SD (*n* = 3). ^#^ *p* < 0.05, ^##^ *p* < 0.01 compared with the control group; *p < 0.05, **p < 0.01 compared with the vehicle group. *PPPs-L*, low-dose PPPs; *PPPs -H*, high-dose PPPs.

**FIGURE 6:**
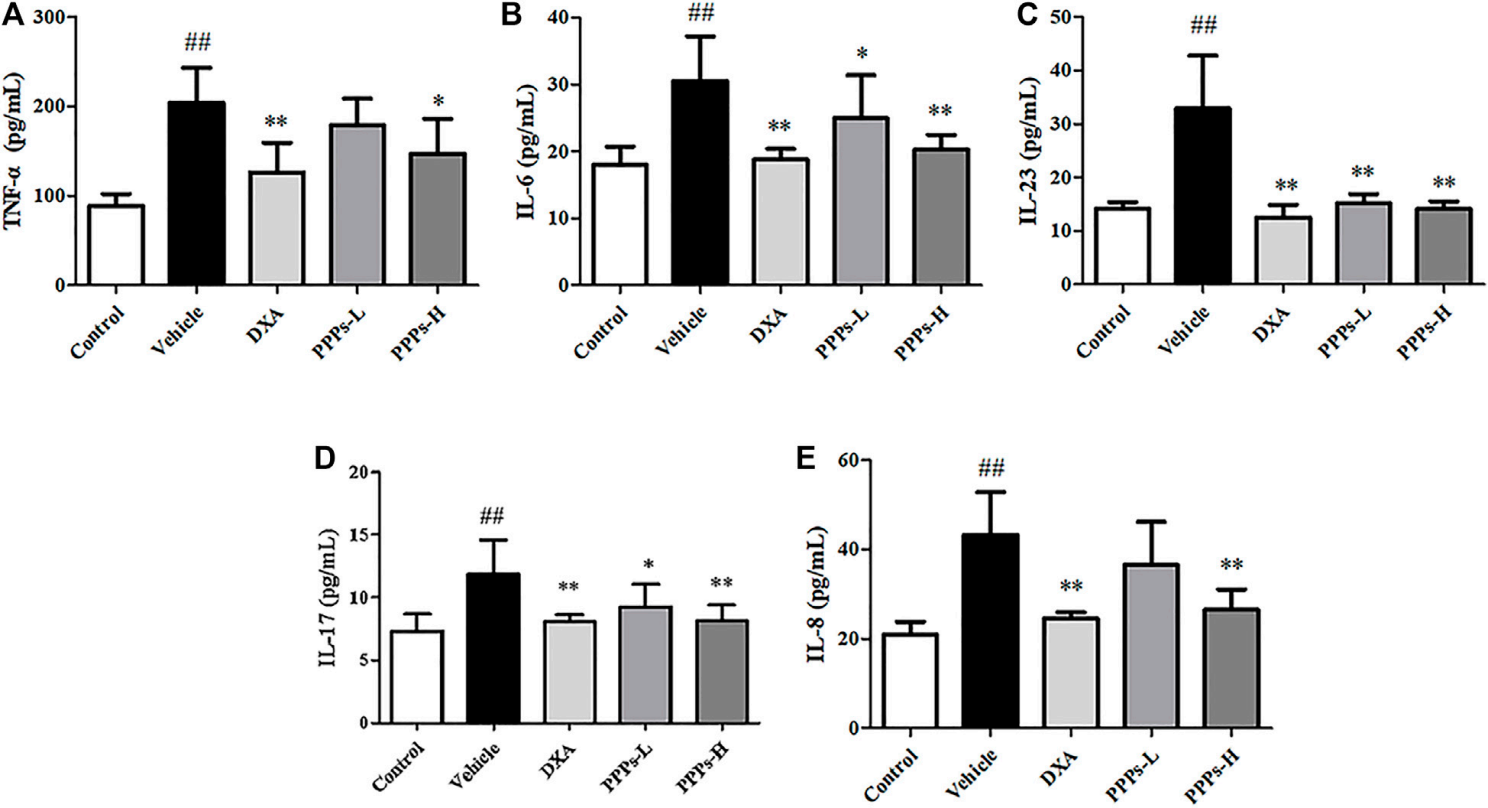
*Punica granatum* peel polysaccharides (PPPs) decreased the levels of inflammatory mediators in mice with imiquimod (IMQ)-elicited psoriasis. The levels of the inflammatory mediators TNF-α **(A)**, IL-6 **(B)**, IL-23 **(C)**, IL-17 **(D)**, and IL-8 **(E)** were measured by ELISA in the serum of IMQ-induced psoriasis-like mice. Data are shown as the mean ± SD (*n* = 6). ^#^ *p* < 0.05, ^##^ *p* < 0.01 compared with the control group; *p < 0.05, **p < 0.01 compared with the vehicle group. *PPPs-L*, low-dose PPPs; *PPPs-H*, high-dose PPPs.

Multiple inflammatory cytokines could be generated by macrophages and dendritic cells in psoriasis (Shao et al., 2016). Therefore, we analyzed the expressions of pro-inflammatory cytokines (TNF-α, IL-6, IL-1β, IL-8, IL-17, and IL-23) in the skin tissue using RT-PCR and in serum using ELISA. As shown in Figures 5, 6, the expressions of these inflammatory cytokines in vehicle mice were markedly improved compared to those in control mice, whereas there were remarkable reductions in the expressions of these inflammatory cytokine in the group treated with PPPs. These results indicated that PPPs could effectively reduce psoriasis-related inflammatory factors.

### *P. granatum* Peel Polysaccharides Suppress the Expressions of Aquaporin-3 and Filaggrin in Imiquimod-Treated Psoriatic Mice

Decreased levels of AQP3 and FLG are commonly seen in skin barrier disruption, which appears to be a key player in epidermal biology in psoriasis (Proksch et al., 2008; Varma et al., 2019). Hence, we determined the expressions of AQP3 and FLG *via* RT-PCR. As shown in Figures 5D, E, the mRNA expressions of AQP3 and FLG were greatly decreased in the vehicle group. However, these two parameters were obviously increased by treatment with PPPs. These results indicated that PPPs could repair the skin barrier induced by topical intervention of IMQ.

### *P. granatum* Peel Polysaccharides Suppress the NF-κB and STAT3 Signaling Pathways in Skin With Psoriasis-Like Lesions

**FIGURE 7:**
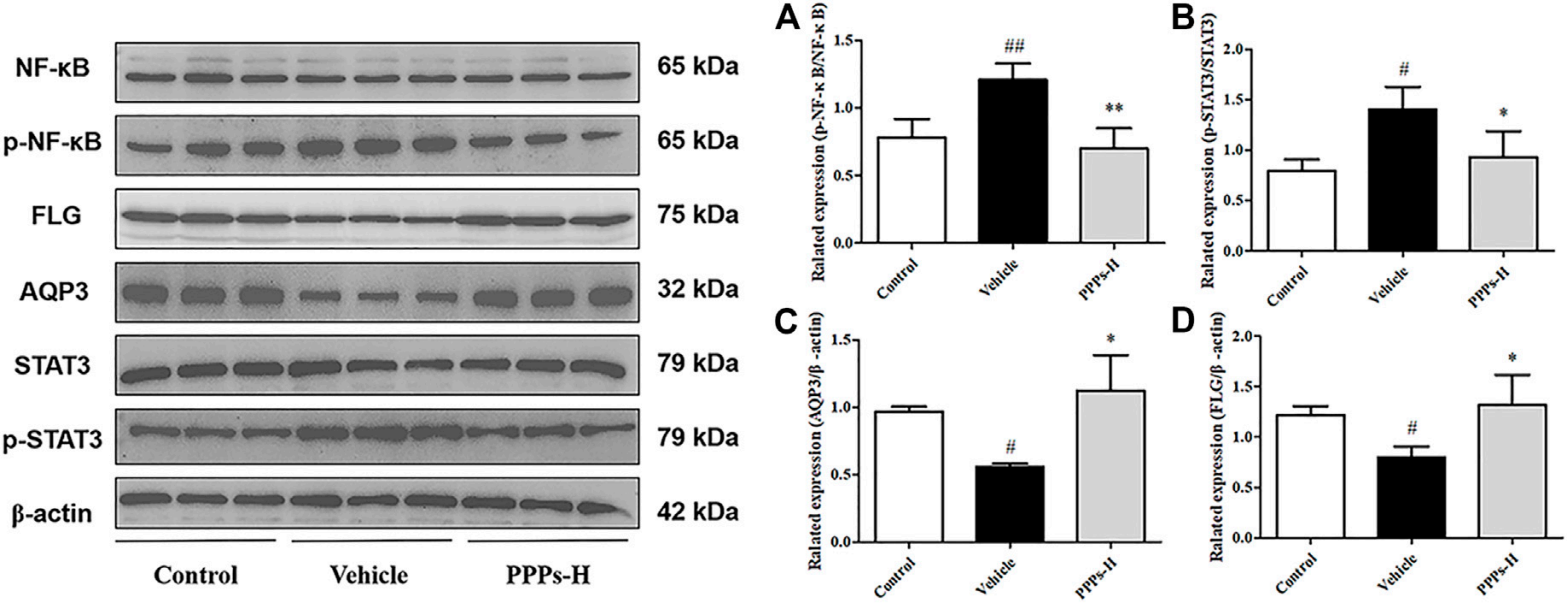
*Punica granatum* peel polysaccharides (PPPs) suppressed aquaporin-3 (AQP3), filaggrin (FLG), and the NF-κB and STAT3 signaling pathways in skin with psoriatic lesions. Effect of PPPs on the translational expressions of p-NF-κB, NF-κB, p-STAT3, STAT3, AQP3, and FLG in the skin samples of mice with imiquimod (IMQ)-elicited psoriasis as determined by Western blotting post-treatment with PPPs. Densitometry analyses of the immunoblotting are shown as p-NF-κB/NF-κB **(A)**, p-STAT3/STAT3 **(B)**, AQP3/β-actin **(C)**, and p-STAT3/β-actin **(D)**. Data are shown as the mean ± SD (*n* = 3). ^#^ *p* < 0.05, ^##^ *p* < 0.01 *vs*. the control group; *p < 0.05, **p < 0.01 *vs*. the vehicle group. *PPPs-L*, low-dose PPPs; *PPPs-H*, high-dose PPPs.

Since the expressions of the pro-inflammatory cytokines (AQP3 and FLG) were suppressed by PPPs in IMQ-induced psoriasis, the underlying mechanism of their anti-psoriatic effects was further explored. Previous evidence has shown that both the NF-κB and STAT3 signaling pathways were overexpressed and activated in skin tissues with psoriasis (Wang et al., 2019). Therefore, Western blotting assay was carried out to explore the potential effect of PPPs on the protein expressions of p-NF-κB, NF-κB, p-STAT3, STAT3, AQP3, and FLG. As shown in Figure 7, the expressions of p-NF-κB and p-STAT3 were upregulated in the skin of psoriatic mice, in contrast to those in the control group. Treatment with PPPs effectively declined the phosphorylation of NF-κB and STAT3. On the other hand, PPPs also enhanced the expressions of AQP3 and FLG. Based on these results, it was indicated that the beneficial effects of PPPs on mice with IMQ-elicited psoriasis were related to their anti-inflammatory effect and skin barrier protection.

## Discussion

Psoriasis is a chronic and relapsing inflammatory and autoimmune-mediated cutaneous ailment that is highly related to excessive inflammatory cytokine production and destruction of the skin barrier function. Therefore, regulating the skin barrier and reducing skin inflammation represent effective treatment strategies for psoriasis. The treatment for psoriasis has achieved clinical improvement. However, the side effects and economic burden due to the long-term application of synthetic or biological agents still pose a frustrating challenge (Whartenby et al., 2008; Boehncke and Schön, 2015; Sikma et al., 2015). Therefore, more effective and safer agents for psoriasis have been in great demand in the drug discovery field over the decades (Greb et al., 2016; Li et al., 2016).

IMQ, which serves as a ligand for TLR7 and TLR8 and as an effective immune activator, can be topically used to induce psoriasis-like skin lesions in a mouse model. It is the most widely used inducer of psoriasis skin inflammation due to its easy modeling and the same phenotypic and histological characteristics as observed in human psoriasis (van der Fits et al., 2009; Di et al., 2021; Zhou et al., 2021).

In this research, the skin barrier protective effects and anti-inflammatory activity of PPPs were investigated. The results showed that PPPs effectively suppressed the PASI scores and TEWL and inhibited the expressions of CD3 and Ki67 in mice with IMQ-elicited psoriasis compared with those in the vehicle group. Moreover, PPPs were observed to remarkably inhibit the levels of psoriasis-related pro-inflammatory mediators in the serum, namely, IL-17, IL-6, TNF-α, IL-8, and IL-23. PPPs altered the mRNA expressions of the inflammatory cytokines (TNF-α, IL-6, and IL-1β) and improved the expressions of AQP3 and FLG in psoriasis-like mouse skin. In addition, PPPs significantly suppressed the NF-κB and STAT3 pathways and accentuated the expressions of AQP3 and FLG when compared to mice with IMQ-elicited psoriasis without treatment.

The H&E staining results indicated that PPPs efficiently ameliorated the IMQ-elicited epidermal thickening, hyperkeratosis, and dermal inflammatory cell infiltration. Ki67 is deemed as a proliferating marker that is indispensable in cell proliferation, and CD3 is considered as an inflammatory marker that can evaluate the effect on inflammatory cell recruitment in the skin (Herman and Herman, 2016; Song et al., 2021). In our study, immunohistochemistry was carried out to probe the expressions of Ki67 and CD3 in skin tissues. The protein expressions of CD3 and Ki67 were depressed in the skin lesions of PPP-treated mice in comparison to those of the vehicle counterpart, which suggested that PPPs could alleviate IMQ-elicited inflammation and proliferation of keratinocytes.

The skin, which is the outer tissue and the largest organ of the human body, not only has the important function of forming an outside barrier that provides chemical, physical, and biological protection against the external environment but also serves as an effective barrier between the inside and outside barriers to prevent excessive water loss (Proksch et al., 2008; Kasemsarn et al., 2016). Many chronic skin disorders, such as psoriasis, are associated with impaired skin barrier function (Segre, 2006; Proksch, 2018). There is a strong connection between FLG and inflammatory dermatosis like psoriasis due to the key role of FLG in skin barrier function (McAleer and Irvine, 2013; Yosipovitch et al., 2019).

Accumulating pieces of evidence have shown that the water channel AQP3, which plays an important role in various skin diseases, is the most abundant aquaporin in the outer epithelial layer of the skin (Patel et al., 2017). Several reports have shown that the levels of FLG and AQP3 were decreased in psoriatic lesions (Voss et al., 2011; Lee et al., 2012; Seleit et al., 2015). Our results revealed that the levels of FLG and AQP3 in psoriatic-like skin were markedly decreased compared to those in the control skin. In contrast, the FLG and AQP3 contents increased simultaneously after PPP treatment compared to those in the vehicle group. These results indicated that PPPs displayed potentially beneficial effect on the skin barrier in IMQ-elicited psoriasis-like mice.

The increased production of pro-inflammatory mediators and chemokines such as IL-1, IL-6, IL-23, IL-17, and TNF-α plays a crucial role in the pathogenesis of psoriasis (Croxford et al., 2014; Grine et al., 2015; Harden et al., 2015; Kim and Krueger, 2015). Previous reports have already revealed that topical administration of IMQ cream on the back of mice stimulated the generation of pro-inflammatory cytokines and chemokines (Di et al., 2021; Song et al., 2021). It has been found that the phosphorylation of STAT3 was activated in psoriatic lesions. The IL-23/Th17 pathway was critical for inflammation in the pathogenesis of psoriasis, which has been demonstrated to have a close relationship with STAT3 activation (Andrés et al., 2013). The NF-κB pathway performed a crucial role in the development of psoriasis, which activated molecular patterns and promoted histological hallmarks (Andrés et al., 2013). Growing evidence has shown that both STAT3 and NF-κB were activated and overexpressed in both psoriasis patients and psoriasis-like mice (Sano et al., 2005; Andres-Ejarque et al., 2021; Yang et al., 2021). In this work, PPPs were found to inhibit the phosphorylation of STAT3 and NF-κB and to downregulate the expressions of pro-inflammatory mediators both in serum and skin lesions of psoriatic mice.

## Conclusion

Overall, this study indicated that PPPs ameliorated the IMQ-elicited inflammation in psoriatic mice *via* restoring the impaired skin barrier function by elevating the expressions of FLG and AQP3 and suppressing the pro-inflammatory cytokines by modulating the STAT3 and NF-κB pathways. PPPs might have the potential to be further developed into a promising therapeutic option for the treatment of psoriasis.

## Data Availability

The original contributions presented in the study are included in the article/Supplementary Material, further inquiries can be directed to the corresponding authors.

## Abbreviations

AQP3, aquaporin-3; FLG, filaggrin; IMQ, imiquimod; NF-κB, nuclear factor-κB; PASI, Psoriasis Area and Severity Index; PPPs, *P. granatum* peel polysaccharides; TEWL, transepidermal water loss.

## References

[B1] Agren J. Zelenin S. Svensson L. B. Nejsum L. N. Nielsen S. Aperia A. (2010). Antenatal Corticosteroids and Postnatal Fluid Restriction Produce Differential Effects on AQP3 Expression, Water Handling, and Barrier Function in Perinatal Rat Epidermis. Dermatol. Res. Pract. 2010, 789729. 10.1155/2010/789729 21234324 PMC3018650

[B2] Alyassin M. Campbell G. M. Masey O'neill H. Bedford M. R. (2020). Simultaneous Determination of Cereal Monosaccharides, Xylo- and Arabinoxylo-Oligosaccharides and Uronic Acids Using HPAEC-PAD. Food Chem. 315, 126221. 10.1016/j.foodchem.2020.126221 32000077

[B3] Andrés R. M. Montesinos M. C. Navalón P. Payá M. Terencio M. C. (2013). NF-κB and STAT3 Inhibition as a Therapeutic Strategy in Psoriasis: *In Vitro* and *In Vivo* Effects of BTH. J. Invest. Dermatol. 133, 2362–2371. 10.1038/jid.2013.182 23594598

[B4] Andres-Ejarque R. Ale H. B. Grys K. Tosi I. Solanky S. Ainali C. (2021). Enhanced NF-Κb Signaling in Type-2 Dendritic Cells at Baseline Predicts Non-response to Adalimumab in Psoriasis. Nat. Commun. 12, 4741. 10.1038/s41467-021-25066-9 34362923 PMC8346545

[B5] Boehncke W.-H. Schön M. P. (2015). Psoriasis. The LancetLancet 386, 983–994. 10.1016/s0140-6736(14)61909-7 26025581

[B6] Chen H. Liu H. Tang B. Chen Y. Han L. Yu J. (2020). The Protective Effects of 18*β*-Glycyrrhetinic Acid on Imiquimod-Induced Psoriasis in Mice via Suppression of mTOR/STAT3 Signaling. J. Immunol. Res. 2020, 1980456. 10.1155/2020/1980456 32908937 PMC7474397

[B7] Chen Y. Zhang Q. Liu H. Lu C. Liang C. L. Qiu F. (2018). Esculetin Ameliorates Psoriasis-like Skin Disease in Mice by Inducing CD4+Foxp3+ Regulatory T Cells. Front. Immunol. 9, 2092. 10.3389/fimmu.2018.02092 30258447 PMC6143660

[B8] Cheong K. L. Wu D. T. Zhao J. Li S. P. (2015). A Rapid and Accurate Method for the Quantitative Estimation of Natural Polysaccharides and Their Fractions Using High Performance Size Exclusion Chromatography Coupled with Multi-Angle Laser Light Scattering and Refractive index Detector. J. Chromatogr. A. 1400, 98–106. 10.1016/j.chroma.2015.04.054 25990349

[B9] Croxford A. L. Karbach S. Kurschus F. C. Wörtge S. Nikolaev A. Yogev N. (2014). IL-6 Regulates Neutrophil Microabscess Formation in IL-17A-driven Psoriasiform Lesions. J. Invest. Dermatol. 134, 728–735. 10.1038/jid.2013.404 24067382

[B10] Deng Y. Han B. X. Hu D. J. Zhao J. Li S. P. (2018). Qualitation and Quantification of Water Soluble Non-starch Polysaccharides from Pseudostellaria Heterophylla in China Using Saccharide Mapping and Multiple Chromatographic Methods. Carbohydr. Polym. 199, 619–627. 10.1016/j.carbpol.2018.06.063 30143170

[B11] Di T. Zhai C. Zhao J. Wang Y. Chen Z. Li P. (2021). Taxifolin Inhibits Keratinocyte Proliferation and Ameliorates Imiquimod-Induced Psoriasis-like Mouse Model via Regulating Cytoplasmic Phospholipase A2 and PPAR-γ Pathway. Int. Immunopharmacol 99, 107900. 10.1016/j.intimp.2021.107900 34233233

[B12] Greb J. E. Goldminz A. M. Elder J. T. Lebwohl M. G. Gladman D. D. Wu J. J. (2016). Psoriasis. Nat. Rev. Dis. Primers 2, 16082. 10.1038/nrdp.2016.82 27883001

[B13] Grine L. Dejager L. Libert C. Vandenbroucke R. E. (2015). An Inflammatory triangle in Psoriasis: TNF, Type I IFNs and IL-17An Inflammatory triangle in Psoriasis: TNF, Type I IFNs and IL-17. Cytokine Growth Factor. Reviewscytokine Growth Factor. Rev. 26, 25–33. 10.1016/j.cytogfr.2014.10.009 25434285

[B14] Harden J. L. Krueger J. G. Bowcock A. M. (2015). The Immunogenetics of Psoriasis: A Comprehensive Review. J. Autoimmun. 64, 66–73. 10.1016/j.jaut.2015.07.008 26215033 PMC4628849

[B15] Herman A. Herman A. P. (2016). Topically Used Herbal Products for the Treatment of Psoriasis - Mechanism of Action, Drug Delivery, Clinical Studies. Planta Med. 82, 1447–1455. 10.1055/s-0042-115177 27574899

[B16] Kasemsarn P. Bosco J. Nixon R. L. (2016). The Role of the Skin Barrier in Occupational Skin Diseases. Curr. Probl. Dermatol. 49, 135–143. 10.1159/000441589 26844905

[B17] Kim J. Krueger J. G. (2015). The Immunopathogenesis of Psoriasis. Dermatol. Clin. 33, 13–23. 10.1016/j.det.2014.09.002 25412780

[B18] Lee Y. Je Y. J. Lee S. S. Li Z. J. Choi D. K. Kwon Y. B. (2012). Changes in Transepidermal Water Loss and Skin Hydration According to Expression of Aquaporin-3 in Psoriasis. Ann. Dermatol. 24, 168–174. 10.5021/ad.2012.24.2.168 22577267 PMC3346907

[B19] Li G. B. Ma S. Yang L. L. Ji S. Fang Z. Zhang G. (2016). Drug Discovery against Psoriasis: Identification of a New Potent FMS-like Tyrosine Kinase 3 (FLT3) Inhibitor, 1-(4-((1h-Pyrazolo[3,4-D]pyrimidin-4-Yl)oxy)-3-Fluorophenyl)-3-(5-(tert-Butyl)isoxazol-3-Yl)urea, that Showed Potent Activity in a Psoriatic Animal Model. J. Med. Chem. 59, 8293–8305. 10.1021/acs.jmedchem.6b00604 27535613

[B20] Li L. Zhang H. Y. Zhong X. Q. Lu Y. Wei J. Li L. (2020). PSORI-CM02 Formula Alleviates Imiquimod-Induced Psoriasis via Affecting Macrophage Infiltration and Polarization. Life Sci. 243, 117231. 10.1016/j.lfs.2019.117231 31887296

[B21] Lu Y. Yang Y. Zhang J. Zhang H. Ma C. Tang X. (2021a). Anti-Angiogenic Efficacy of PSORI-CM02 and the Associated Mechanism in Psoriasis *In Vitro* and *In Vivo* . Front. Immunol. 12. 10.3389/fimmu.2021.649591 PMC8119787 33995368

[B22] Lu Y. Yang Y. Zhang J. Zhang H. Ma C. Tang X. (2021b). Anti-Angiogenic Efficacy of PSORI-CM02 and the Associated Mechanism in Psoriasis *In Vitro* and *In Vivo* . Front. Immunol. 12, 649591. 10.3389/fimmu.2021.649591 33995368 PMC8119787

[B23] Lv H. Liu X. Chen W. Xiao S. Ji Y. Han X. (2021). Yangxue Jiedu Fang Ameliorates Psoriasis by Regulating Vascular Regression via Survivin/PI3K/Akt Pathway. J. Immunol. Res. 2021, 4678087. 10.1155/2021/4678087 33532507 PMC7834796

[B24] Mahil S. K. Smith C. H. (2019). Psoriasis Biologics: a new era of Choice. Lancet 394, 807–808. 10.1016/s0140-6736(19)31772-6 31402113

[B25] Mcaleer M. A. Irvine A. D. (2013). The Multifunctional Role of Filaggrin in Allergic Skin Disease. J. Allergy Clin. Immunol. 131, 280–291. 10.1016/j.jaci.2012.12.668 23374260

[B26] Michalek I. M. Loring B. John S. M. (2017). A Systematic Review of Worldwide Epidemiology of Psoriasis. J. Eur. Acad. Dermatol. Venereol. 31, 205–212. 10.1111/jdv.13854 27573025

[B27] Patel R. Kevin Heard L. Chen X. Bollag W. B. (2017). Aquaporins in the Skin. Adv. Exp. Med. Biol. 969, 173–191. 10.1007/978-94-024-1057-0_11 28258573

[B28] Proksch E. Brandner J. M. Jensen J. M. (2008). The Skin: an Indispensable Barrier. Exp. Dermatol. 17, 1063–1072. 10.1111/j.1600-0625.2008.00786.x 19043850

[B29] Proksch E. (2018). pH in Nature, Humans and Skin. J. Dermatol. 45, 1044–1052. 10.1111/1346-8138.14489 29863755

[B30] Sano S. Chan K. S. Carbajal S. Clifford J. Peavey M. Kiguchi K. (2005). Stat3 Links Activated Keratinocytes and Immunocytes Required for Development of Psoriasis in a Novel Transgenic Mouse Model. Nat. Med. 11, 43–49. 10.1038/nm1162 15592573

[B31] Segre J. A. (2006). Epidermal Barrier Formation and Recovery in Skin Disorders. J. Clin. Invest. 116, 1150–1158. 10.1172/jci28521 16670755 PMC1451215

[B32] Seleit I. Bakry O. A. Al Sharaky D. Ragheb E. (2015). Evaluation of Aquaporin-3 Role in Nonmelanoma Skin Cancer: An Immunohistochemical Study. Ultrastruct. Pathol. 39, 306–317. 10.3109/01913123.2015.1022241 26107428

[B33] Shakhmatov E. G. Makarova E. N. Belyy V. A. (2019). Structural Studies of Biologically Active Pectin-Containing Polysaccharides of Pomegranate Punica Granatum. Int. J. Biol. Macromol 122, 29–36. 10.1016/j.ijbiomac.2018.10.146 30359658

[B34] Shao F. Tan T. Tan Y. Sun Y. Wu X. Xu Q. (2016). Andrographolide Alleviates Imiquimod-Induced Psoriasis in Mice via Inducing Autophagic Proteolysis of MyD88. Biochem. Pharmacol. 115, 94–103. 10.1016/j.bcp.2016.06.001 27265145

[B35] Sikma M. A. Van Maarseveen E. M. Van De Graaf E. A. Kirkels J. H. Verhaar M. C. Donker D. W. (2015). Pharmacokinetics and Toxicity of Tacrolimus Early after Heart and Lung Transplantation. Am. J. Transpl. 15, 2301–2313. 10.1111/ajt.13309 26053114

[B36] Song C. Yang C. Meng S. Li M. Wang X. Zhu Y. (2021). Deciphering the Mechanism of Fang-Ji-Di-Huang-Decoction in Ameliorating Psoriasis-like Skin Inflammation via the Inhibition of IL-23/Th17 Cell axis. J. Ethnopharmacol 281, 114571. 10.1016/j.jep.2021.114571 34464701

[B37] Templeton D. W. Quinn M. Van Wychen S. Hyman D. Laurens L. M. (2012). Separation and Quantification of Microalgal Carbohydrates. J. Chromatogr. A. 1270, 225–234. 10.1016/j.chroma.2012.10.034 23177152

[B38] Van Der Fits L. Mourits S. Voerman J. S. Kant M. Boon L. Laman J. D. (2009). Imiquimod-induced Psoriasis-like Skin Inflammation in Mice Is Mediated via the IL-23/IL-17 axis. J. Immunol. 182, 5836–5845. 10.4049/jimmunol.0802999 19380832

[B39] Varma S. R. Sivaprakasam T. O. Arumugam I. Dilip N. Raghuraman M. Pavan K. B. (2019). *In Vitro* anti-inflammatory and Skin Protective Properties of Virgin Coconut Oil. J. Tradit Complement. Med. 9, 5–14. 10.1016/j.jtcme.2017.06.012 30671361 PMC6335493

[B40] Voss K. E. Bollag R. J. Fussell N. By C. Sheehan D. J. Bollag W. B. (2011). Abnormal Aquaporin-3 Protein Expression in Hyperproliferative Skin Disorders. Arch. Dermatol. Res. 303, 591–600. 10.1007/s00403-011-1136-x 21400035 PMC3752693

[B41] Wang A. Wei J. Lu C. Chen H. Zhong X. Lu Y. (2019). Genistein Suppresses Psoriasis-Related Inflammation through a STAT3-NF-κb-dependent Mechanism in Keratinocytes. Int. Immunopharmacol 69, 270–278. 10.1016/j.intimp.2019.01.054 30743203

[B42] Wang X. Xu Y. Lian Z. Yong Q. Yu S. (2014). A One-step Method for the Simultaneous Determination of Five Wood Monosaccharides and the Corresponding Aldonic Acids in Fermentation Broth Using High-Performance Anion-Exchange Chromatography Coupled with a Pulsed Amperometric Detector. J. Wood Chem. Tech. 34, 67–76. 10.1080/02773813.2013.838268

[B43] Whartenby K. A. Small D. Calabresi P. A. (2008). FLT3 Inhibitors for the Treatment of Autoimmune Disease. Expert Opin. Investig. Drugs 17, 1685–1692. 10.1517/13543784.17.11.1685 PMC4882767 18922105

[B44] Wu Y. Zhu C. P. Zhang Y. Li Y. Sun J. R. (2019). Immunomodulatory and Antioxidant Effects of Pomegranate Peel Polysaccharides on Immunosuppressed Mice. Int. J. Biol. Macromol 137, 504–511. 10.1016/j.ijbiomac.2019.06.139 31229542

[B45] Yang Z. Chen Z. Wang C. Huang P. Luo M. Zhou R. (2021). STAT3/SH3PXD2A-AS1/miR-125b/STAT3 Positive Feedback Loop Affects Psoriasis Pathogenesis via Regulating Human Keratinocyte Proliferation. Cytokine 144, 155535. 10.1016/j.cyto.2021.155535 33994260

[B46] Yao D. N. Lu C. J. Wen Z. H. Yan Y. H. Lu L. M. Wu H. M. (2021). Comparison of PSORI-CM01 Granules and Yinxieling Tablets for Patients with Chronic Plaque Psoriasis: a Pilot Study for a Randomized, Double-Blinded, Double-Dummy, Multicentre Trial. Ann. Palliat. Med. 10, 2036–2047. 10.21037/apm-20-2575 33549019

[B47] Yosipovitch G. Misery L. Proksch E. Metz M. Ständer S. Schmelz M. (2019). Skin Barrier Damage and Itch: Review of Mechanisms, Topical Management and Future Directions. Acta Derm Venereol. 99, 1201–1209. 10.2340/00015555-3296 31454051

[B48] Zhai X. Zhu C. Li Y. Zhang Y. Duan Z. Yang X. (2018a). Optimization for Pectinase-Assisted Extraction of Polysaccharides from Pomegranate Peel with Chemical Composition and Antioxidant Activity. Int. J. Biol. Macromol 109, 244–253. 10.1016/j.ijbiomac.2017.12.064 29242125

[B49] Zhai X. Zhu C. Zhang Y. Sun J. Alim A. Yang X. (2018b). Chemical Characteristics, Antioxidant Capacities and Hepatoprotection of Polysaccharides from Pomegranate Peel. Carbohydr. Polym. 202, 461–469. 10.1016/j.carbpol.2018.09.013 30287023

[B50] Zhou T. Zhang S. Zhou Y. Lai S. Chen Y. Geng Y. (2021). Curcumin Alleviates Imiquimod-Induced Psoriasis in Progranulin-Knockout Mice. Eur. J. Pharmacol. 909, 174431. 10.1016/j.ejphar.2021.174431 34428436

[B51] Zhou W. Hu M. Zang X. Liu Q. Du J. Hu J. (2020). Luteolin Attenuates Imiquimod-Induced Psoriasis-like Skin Lesions in BALB/c Mice via Suppression of Inflammation Response. Biomed. Pharmacother. 131, 110696. 10.1016/j.biopha.2020.110696 32920513

