# Supplementary figures and images for: P. granatum Peel Polysaccharides Ameliorate Imiquimod-Induced Psoriasis-Like Dermatitis in Mice via Suppression of NF-κB and STAT3 Pathways
Source: Front Pharmacol. 2022 Jan 28;12:806844. doi: 10.3389/fphar.2021.806844 (PMC8831316; doi:10.3389/fphar.2021.806844)

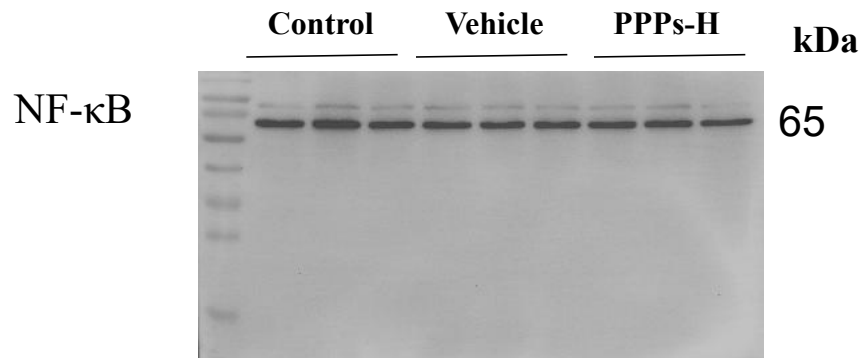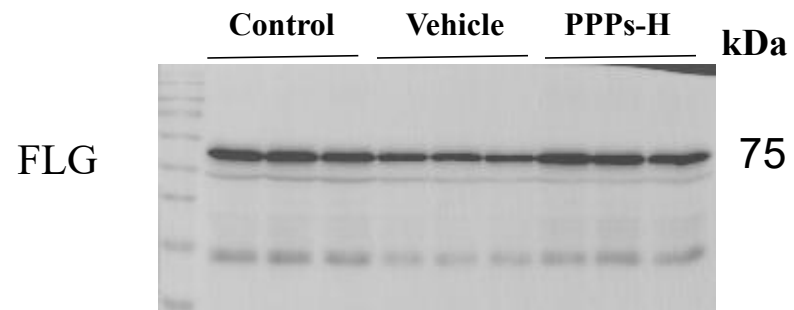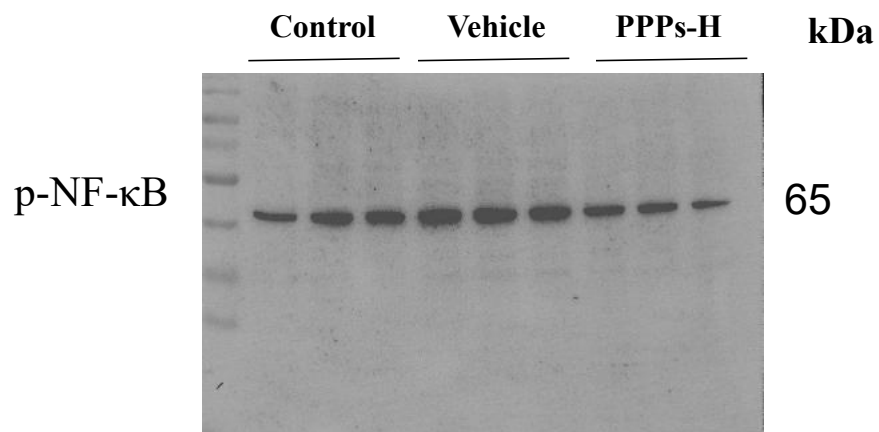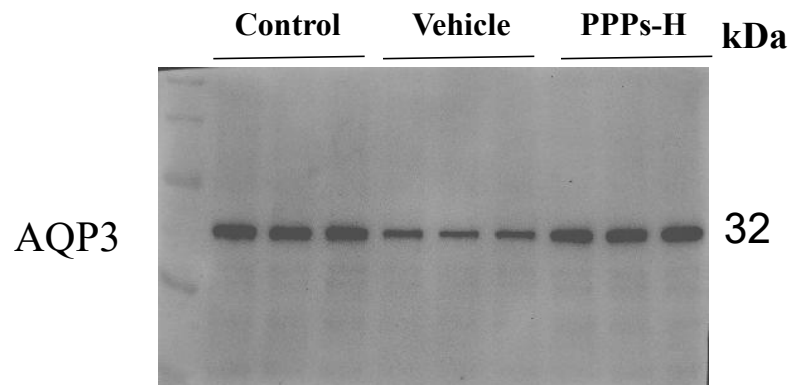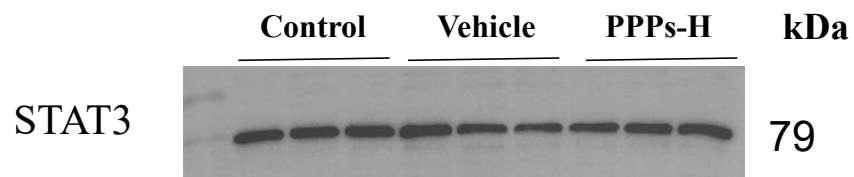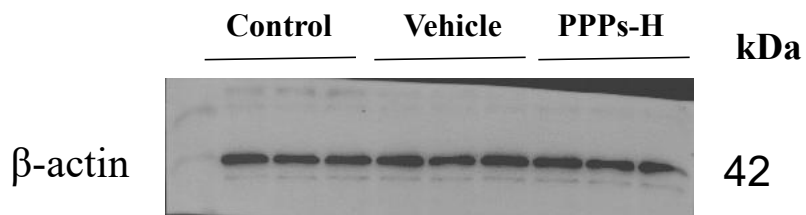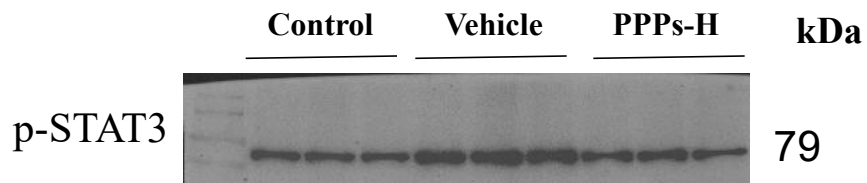

Supplement: Supplementary file 2 [file DataSheet3.PDF]

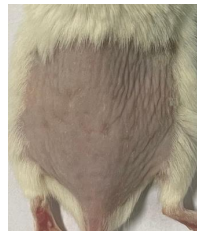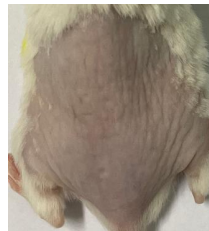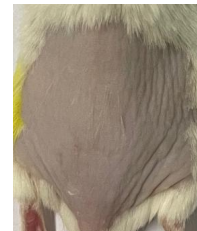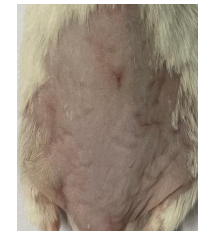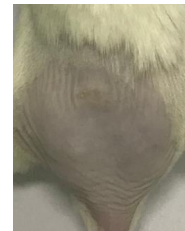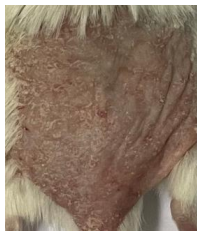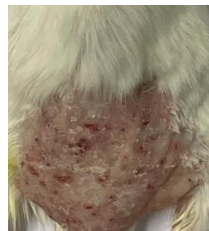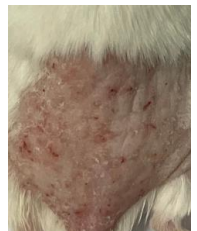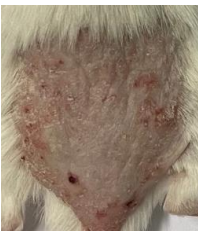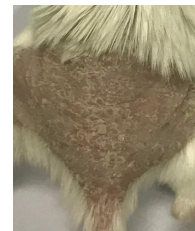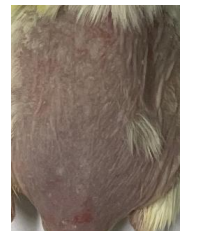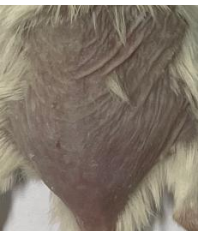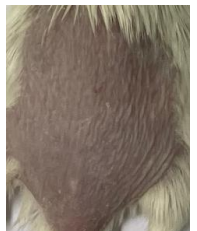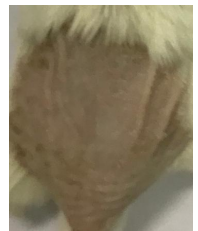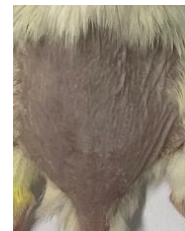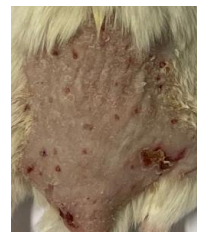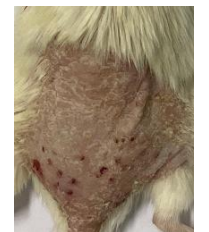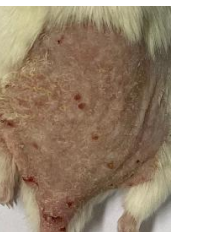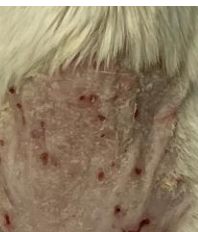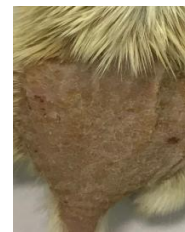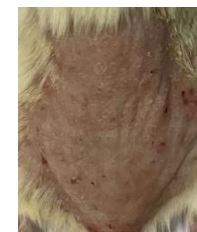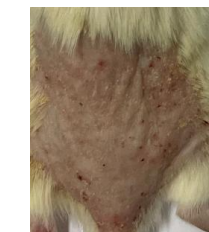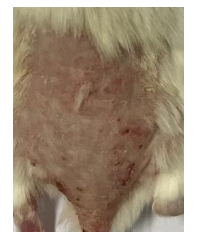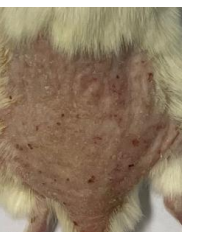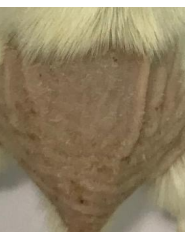

Supplement: Supplementary file 3 [file DataSheet1.PDF]
